# Meaning attribution in the West African green monkey: influence of call type and context

**DOI:** 10.1007/s10071-013-0660-9

**Published:** 2013-07-12

**Authors:** Tabitha Price, Julia Fischer

**Affiliations:** 1 Cognitive Ethology Lab, German Primate Center, Göttingen, Germany; 2 Courant Research Centre for the Evolution of Social Behaviour, Georg August University of Göttingen, Göttingen, Germany

**Keywords:** Alarm call, *Chlorocebus sabaeus*, Language evolution, Pragmatics, Referential, Vocal communication

## Abstract

The search for the evolutionary roots of human language has fuelled much research into the cognitive mechanisms underlying communication in nonhuman animals. One core issue has been whether the context-specific calls of nonhuman animals are meaningful, with call meaning inferred from recipients’ responses in the absence of supporting contextual cues. This direct inference may well offer an oversimplified view of how vocalisations are perceived, however, as responses under natural conditions are likely guided by contextual cues as well as by the signal. In this study, we investigate how the anti-predator responses of green monkeys, *Chlorocebus sabaeus*, are affected by alarm call structure and by context. We first simulated the presence of leopards and snakes to elicit alarm vocalisations and to identify predator-typical response behaviours. In both contexts, the monkeys produced chirp calls that revealed only graded variation in relation to predator type. We then carried out playback experiments to explore whether green monkeys would respond with predator-typical behaviour to leopard and snake chirps, and whether contextual cues, in the form of pre-exposure to a leopard or snake model, would modify these responses. Irrespective of context, subjects were more likely to respond to leopard chirps with a leopard-typical response. Predator priming did not have a significant effect on the type of response, but, together with call type, did affect response duration. This suggests that the immediate attribution of meaning was influenced by acoustic cues, whilst receiver’s prior knowledge was incorporated to guide subsequent behaviour.

**Electronic supplementary material:**

The online version of this article (doi:10.1007/s10071-013-0660-9) contains supplementary material, which is available to authorized users.

## Introduction

What do the vocalisations of animals mean? This question is central to the debate regarding the similarities and differences between nonhuman animal (hereafter animal) communication and human language, and consequently, language evolution. The finding that vervet monkeys (*Chlorocebus pygerythrus*) produce predator-specific alarm calls that elicit appropriate response behaviours even in the absence of contextual cues led initially to claims that these calls possessed semantic properties (Seyfarth et al. 1980a). The general consensus that, within animal communication, signallers and receivers do not share a representational state and are not motivated to communicate as a result of attributing mental states to one another (Cheney and Seyfarth 1992a; Rendall et al. 2009) implies, however, that animal vocalisations are not meaningful in the linguistic sense of the word (Cheney and Seyfarth 1992b; Rendall et al. 2009; Scarantino 2010).

Over the last 20 years, signals that are elicited only by stimuli belonging to a common category (i.e. are context specific) and that cause signal receivers to respond with stimulus-appropriate behaviours even in the absence of contextual cues have been termed “functionally referential” (Marler et al. 1992; Macedonia and Evans 1993). This terminology was meant to emphasise that such signals are “not exactly like human words, but rather appear to function in the same way” (Hauser 1997 p. 509). Numerous studies indicate that receiver responses cannot be explained only in terms of unconditioned reactions to the acoustic properties of a call (reviewed in Seyfarth et al. 2010) or by perceptual similarities between the call and the stimulus (Zuberbühler et al. 1999). Instead, across a broad array of taxa, signal receivers respond to calls as if they had learnt to associate them with a specific predator class (Manser et al. 2001; Gill and Sealy 2004; Kirchhof and Hammerschmidt 2006), degree of risk (Furrer and Manser 2009), food (Evans and Evans 2007), social situation (Faragó et al. 2010) and/or individual (Cheney and Seyfarth 1982; Vignal et al. 2008). It is worth noting, however, that this is not a universal property of calls. The alarm calls of American red squirrels, for example, demonstrate low predator specificity (Digweed and Rendall 2009), and the recruitment calls of the banded mongoose convey information about the risk posed by a stimulus rather than stimulus type (Furrer and Manser 2009). In addition, whilst the vocalisations of many species are structurally discrete, this is not a pre-requisite for functional reference; context-specific calls that differ along a graded continuum may also elicit appropriate responses from signal receivers in the absence of supporting contextual cues (Fischer 1998), although this ability may require a degree of learning (Fischer et al. 2000).

The above description of receivers associating calls with referents is in line with insights into learning theory and more specifically Pavlovian conditioning (reviewed in Rescorla 1988), whereby functionally referential alarm calls can be classified as a conditioned stimulus (Seyfarth and Cheney 2003) with an indexical relationship between the call and referent (reviewed in Wheeler and Fischer 2012). But whilst laboratory experiments within the framework of learning theory have shown effects of context specificity on the initial formation, extinction and renewal of conditioned responses in humans and other animals (Bouton et al. 2006; Huff et al. 2011), and identified neurological mechanisms underlying these effects (Hobin et al. 2003), the current definition of functional reference requires the attribution of meaning in the absence of relevant contextual cues. An alternative proposal in keeping with the influence of context on meaning attribution is that context specificity is not a requirement for calls to function referentially, only that the less referentially specific a call is, the more important contextual cues will be for an accurate attribution of meaning (Scarantino in press; Wheeler and Fischer 2012). In this study, we therefore use meaning to refer to what the signal receiver infers from a signal, for example the presence of an external stimulus or the subsequent behaviour of the signaller.

Studies of animal communication have shown that the response behaviours of signal receivers are, in some cases, modified by contextual cues, including the signal receiver’s prior experience (Zuberbühler 2000; Engh et al. 2006; Akçay et al. 2009; Arnold and Zuberbühler 2013), and contextual cues at the time of hearing a call (Wheeler and Hammerschmidt in press; Rendall et al. 1999), which may include the presence or absence of additional signals (e.g. multimodal signals; reviewed in Partan and Marler 1999). But despite this, and the fact that the role of context on call perception presents a possible parallel with pragmatics in human language (Scott-Phillips 2009; Wheeler et al. 2011), we know little about how context specificity and structure (discrete versus graded) of a call affect the degree to which contextual cues are incorporated.

More than 40 years have gone by since Struhsaker (1967) described the vervet monkey’s predator-specific alarm calls, and they remain the classic example of functional reference within the animal kingdom. However, a relatively high number of individuals did not respond appropriately to alarm calls when they were broadcast in the absence of supporting contextual cues (Seyfarth et al. 1980b), and chirps are described as being produced in response to both avian and major terrestrial predators (Struhsaker 1967). Taken together, it seems likely that both context and call structure contribute to the attribution of call meaning by conspecifics.

Like adult female vervets, adult female green monkeys (*C. sabaeus*) produce chirp calls in response to more than one predator class. The green monkey is a close relative of the vervet, and they were previously classified as conspecifics (Napier 1981).We here follow the taxonomy of Groves (2001), however, which places the green monkey as a closely related congener to the vervet. In the case of green monkeys, females produce chirp calls to both snake and leopard models (hereafter referred to as “snake chirps” and “leopard chirps”), and these calls sound acoustically similar to one another. In this study, we first investigated predator-specific behaviours in the green monkeys and analysed the acoustic structure of snake and leopard alarm chirps. We then performed experiments in which subjects were exposed to a predator model (leopard or snake) before playing back a leopard or snake chirp. If chirp calls given to leopards and snakes are strongly referential, they should elicit predator-typical avoidance behaviours irrespective of supporting or conflicting contextual cues. If, however, context also plays a role in how conspecifics’ attribute meaning to these calls, then priming with a corresponding predator model (e.g. priming with a leopard model prior to playing a leopard chirp) should increase the occurrence of predator-typical responses relative to responses elicited by the calls alone, whilst priming with a conflicting predator model (i.e. priming with a snake model prior to playing a leopard chirp) should have the opposite effect.

## Study site and subjects

The study was conducted over two field seasons (January-June 2010 and December 2010–June 2011) within Niokolo Koba National Park in southeast Senegal (13°01′34″N, 13°17′41″W), an area encompassing 913,000 ha of predominantly Sudano-Guinean savannah interspersed with woodland and gallery forest (Frederiksen and Lawesson 1992). Green monkeys are found throughout the park, living in species-typical multi-male multi-female groups (Dunbar 1974). Data were collected in the vicinity of the Simenti Centre de Recherche de Primatologie (CRP Simenti) from four groups of free-ranging green monkeys (“Simenti” 16–21 individuals; “Mare” 12–18 individuals; “Lions” 19–26 individuals; “Niokolo” 27–32 individuals; ranges reflect changes in group size over the duration of the study period). Study subjects were habituated adult males and females that were recognised individually from natural markings on the face and body. Pythons, venomous snakes and leopards were all observed in the vicinity of the field site over the course of the study.

## Behavioural response to terrestrial predators

### Experimental protocol

Vervet monkeys tend to respond to snakes by looking down and standing bipedally, and to leopards by climbing up into trees (Cheney and Seyfarth 1992b). To test whether green monkeys respond to these terrestrial predators with these same predator-typical behaviours, we simulated the presence of snakes and leopards and video-taped their behavioural response. For details of predator simulations and modes of presentation, see Online Resource 1. Subjects were provisioned with peanuts prior to model presentation to position individuals on the ground and to ensure that subject behaviour (stationary feeding) was consistent in the time period preceding all playbacks. Experiments were discarded if the subject moved out of sight within the first 10 s of the experiment (5 cases), if the subject responded to a different stimulus prior to model presentation (3 cases) or if there were technical problems with the equipment (1 case), resulting in a total of 17 leopard model (adult female *n* = 8, adult male *n* = 9) and 19 snake model (adult female *n* = 9, adult male *n* = 10) experiments for analysis.

### Behavioural analysis

Behavioural responses of subjects were filmed using a Sony Handycam (DCR-HC90E), and videos were imported into Adobe Premiere Pro CS4 with a time resolution of 25 frames/second. Frame-by-frame analysis set at five-frame jumps was used to score the subject’s behaviour as one of four mutually exclusive categories (rest, bipedal, terrestrial displacement or arboreal displacement) at 0.2 s intervals for a period of 10 s, starting with the subject’s first response to the predator model. We had initially planned to include looking direction as a behavioural measure, but poor visibility made it impossible to score this reliably from the videos. Maximum height of the subject within 30 s of viewing the model was recorded as 0 m, >0 m but <2 m or >2 m. Because video encoding is susceptible to observer bias, all videos were reanalysed by a second condition-naive observer. Intra-class correlation coefficient (ICC) was 0.986, indicating a high level of inter-observer reliability.

### Statistical analysis

We used a generalised linear mixed model (GLMM) with binomial error structure and logit link function to test whether snake models were more likely than leopard models to elicit bipedal behaviour, with bipedal behaviour scored as absent or present. A second GLMM with Poisson error structure and a log link function was run to test whether leopard models would cause subjects to climb into a tree more often than snake models, with response behaviour scored as one of the three height categories described above. Both GLMMs were run with the type of predator model (snake or leopard) as the fixed effect and subject identity included as a random effect using the function lmer of the lme4 Package (Bates et al. 2011). We used a likelihood ratio test (ANOVA using “Chisq” argument) to compare the full models with a null model (comprising only the intercept and the random effect) in order to calculate the overall effect of the predator model. All models were fitted in R (R Development Core Team 2011).

### Results and discussion

**Fig. 1:**
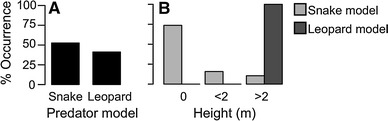
*Bar plots* illustrating subjects’ behavioural responses to snake (*n* = 19) and leopard (*n* = 17) models. **a** The percentage of individuals that stood bipedally within 10 s of seeing a predator model. **b** The maximum height of subjects within 30 s of seeing a predator model

There was no significant difference in the bipedal behaviour of subjects following the presentation of snake and leopard models (likelihood ratio test: *χ* ^*2*^ = 0.47, *df* = 1, *P* = 0.491; Fig. 1a). Like vervet monkeys, green monkeys do sometimes respond to snakes by standing bipedally, but since they also responded to leopard models with bipedal behaviour, this did not constitute a predator-specific response. Whilst vervet monkeys were described as responding with bipedal behaviour to snakes, they responded to playbacks of alarm calls given to both snakes and leopards with bipedal behaviour (Seyfarth et al. 1980b). For vervets and green monkeys, bipedalism may therefore function not only as a mobbing behaviour but also as a form of unspecific vigilance. As we were not able to assess gaze direction, we cannot discount that bipedalism for the purpose of either scanning the ground for snakes, or scanning the horizon for cats, could constitute a predator-specific response. In consequence, from the results described in this section, it is not possible to identify a snake-specific behavioural response with which the referential specificity of snake chirps, with and without contextual cues, could be tested.

Green monkeys, like vervets, were more likely to climb into a tree in response to leopard than snake models (likelihood ratio test: *χ* ^*2*^ = 22.49, *df* = 1, *P* < 0.001, Fig. 1b). In particular, whilst snake models occasionally prompted subjects to jump into trees at <2 m, leopard models always resulted in subjects climbing higher (>2 m) into a tree. This can be explained as an adaptive response, whereby green monkeys, like vervets, are likely safest from leopards high up in the trees (Cheney and Seyfarth 1992b). Thus, it would seem that climbing >2 m into a tree is a more leopard-specific response than simply climbing into a tree.

## Chirp playback stimuli

### Playback stimuli

**Fig. 2:**
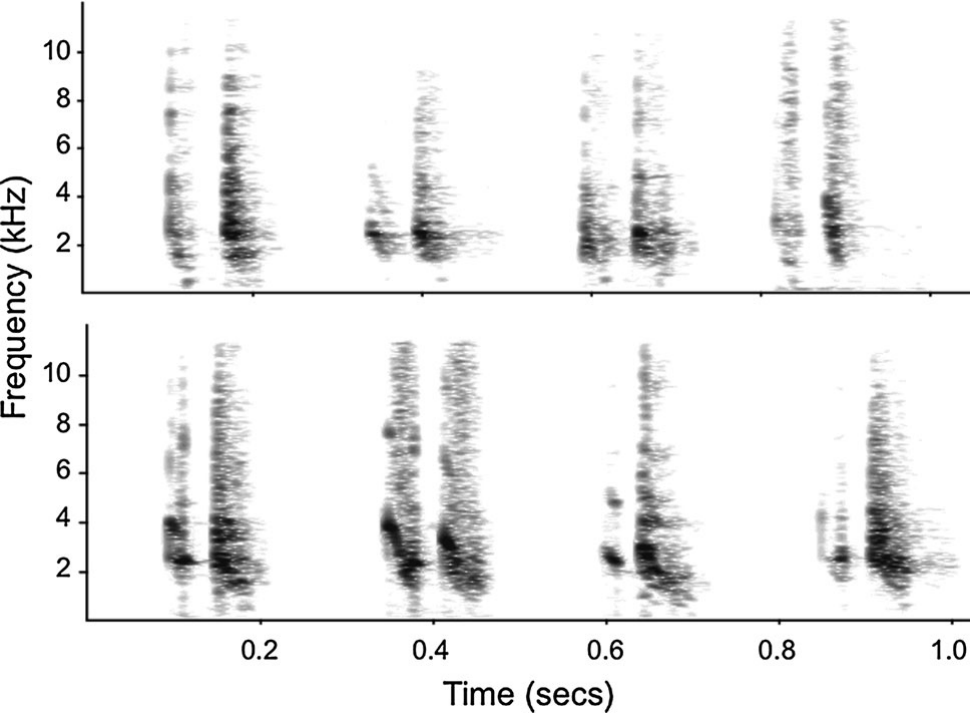
Spectrograms of paired chirp calls given to model snakes (*top row*) and model leopards (*bottom row*). For each context, the calls of four individuals are presented. The same individuals do not contribute calls for both contexts. Spectrograms were made with a 512 FFT and a Hamming window

Alarm chirps used as playback stimuli were elicited by the presentation of leopard and snake models. Calls were recorded from adult females and juveniles from all four study groups using a Marantz PMD661 solid-state recorder (44.1 kHz sampling rate; 16-bit sampling depth) connected to a Sennheiser ME66/K6 directional microphone. Vocal recordings were transferred to a PC, and Avisoft-SASLab Pro (R. Specht Berlin, Germany, version 5.1.20) was used to check recording quality, filter recordings to remove background noise below 0.1 kHz and to prepare the playback stimuli. Each playback sequence was constructed from chirps produced during a single calling bout, although not always in their natural order, as it was sometimes necessary to replace low quality chirps with higher quality exemplars produced later in the calling bout. A total of ten pairs of playback sequences were compiled, whereby each pair consisted of a sequence of chirps given to a leopard model and a sequence of chirps given to a snake model. The number of chirps, inter-call durations, and sequence duration were consistent between paired sequences, all call sequences were normalised to the same maximum volume and inter-call durations were additionally controlled to fall within the range of naturally emitted calls. When possible, the same individual produced both call sequences within a pair, and at all times, call sequences within a pair were produced by a caller from the same social group. With one exception, all leopard chirp and all snake chirp playback stimuli were taken from the calling bouts of different individuals, and in this exception, different calls from the same individual were used to construct two playback sequences. Calls of nonpredatory birds were recorded locally and modified to be of a similar length and volume to chirp sequences for use as control stimuli. To avoid pseudo-replication, a different playback sequence was used for each playback experiment. Spectrograms illustrating snake and leopard chirps are shown in Fig. 2.

### Acoustic analysis

To assess the acoustic structure of chirp calls used as playback stimuli (*N* = 124), Avisoft-SASLab Pro was used to add silent margins and reduce the sampling frequency of single call units to 22.05 kHz. Call units were then transformed in their frequency–time domain using a fast Fourier transformation (FFT) size of 1,024 points, Hamming window and 93.75 % overlap. The resulting frequency–time spectra were analysed with LMA (K. Hammerschmidt, version 2012_9), a custom software sound analysis tool (Schrader and Hammerschmidt 1997). Using Avisoft, duration was extracted from the wav file, and Wiener entropy was calculated; LMA was used to calculate robust acoustic parameters describing energy distribution throughout the call unit. A description of parameters used for analyses are given in Table 1.

**Table 1:** Description of the acoustic parameters used to describe chirp call structure

Measurement	Description
Duration (ms)	Duration of call unit
Peak frequency_1–4 (Hz)	Mean peak frequency at 1st, 2nd, 3rd and 4th temporal quartiles
First quartile_1–4 (Hz)	Mean first frequency quartile at 1st, 2nd, 3rd and 4th temporal quartiles
Second quartile_1–4 (Hz)	Mean second frequency quartile at 1st, 2nd, 3rd and 4th temporal quartiles
Third quartile_1–4 (Hz)	Mean third frequency quartile at 1st, 2nd, 3rd and 4th temporal quartiles
Wiener entropy	Mean value of noise within call. 0 = pure tone, 1 = random noise
Frequency range (Hz)	Mean frequency range
PF jump (Hz)	Maximum difference between successive peak frequencies
Peak frequency deviation (Hz)	Mean deviation between peak frequency and linear trend
Linear trend	Factor of linear trend of peak frequency

### Statistical analysis

To avoid entering correlated parameters into the discriminant function analysis (LDA), a stepwise variable selection with leave-one-out cross-validation (stepclass function of the R-package “klaR”, Weihs et al. 2005) was used to first identify an optimum subset of variables for classification. Acoustic parameters were transformed when necessary to meet test assumptions (Online Resource 1) and then entered into the stepwise classification, with predator type set as the grouping variable. Following this, the selected variables were entered (post z-transformation) into a linear LDA using the lda function of the R-package “mass” (Venables and Ripley 2002), with predator type again set as the grouping variable. A leave-one-out procedure was used to calculate the percentage of calls correctly classified, and a subset of the data (*N* = 93) was entered into a nested permuted discriminant function analysis (pDFA, Mundry and Sommer 2007) to re-calculate classification scores whilst controlling for caller identity.

## Results and discussion

**Fig. 3:**
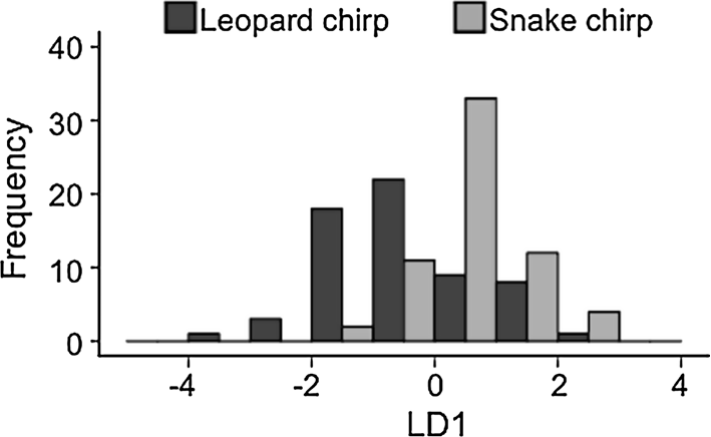
Histogram showing the distribution of the first linear discriminant scores for chirp calls given in response to leopard (*n* = 62) and snake (*n* = 62) models

Stepwise variable selection identified duration and peak frequency_1 as the most important variables for differentiating between chirps produced in response to different predator types. Based on differences in these two variables, LDA (with leave-one out validation) correctly identified leopard and snake chirps in 75 % of cases. A similar result was found using a pDFA on a subset of the calls in order to control for caller identity, with 72 % of calls correctly classified. On the basis of the LDA classification, chirp calls were correctly assigned to the predator type eliciting calling more often than would be expected by chance (Binomial test, chirps *N* = 124, *P* < 0.05), and each snake playback stimulus (with one exception) had a higher mean discriminant score than the leopard playback stimulus with which it was paired. The relatively high number of calls that were incorrectly classified, however, supports the acoustic impression that structural differences between leopard and snake chirps are graded rather than discrete in nature (Fig. 3), suggesting that, for many calls, receivers would be unable to determine whether the signal was indicative of the presence of either a leopard or a snake.

Duration contributed most to distinguishing between leopard and snake chirps, followed by peak frequency_1, with leopard chirps being longer than snake chirps and demonstrating a higher early peak frequency. Studies in a broad array of species suggest that as callers experience an increase in arousal, their vocalisations become longer and higher in frequency (reviewed in Briefer 2012). In line with these findings, the structural differences identified in this study between snake and leopard chirps could be attributed to callers being more aroused in the presence of a leopard than a snake.

This analysis does not allow for conclusions to be drawn about the probability of chirps being produced in the presence of a snake or leopard, or whether chirps are also produced in nonpredator contexts. Results do suggest, however, that the chirp is similar to the graded alarm calls of Barbary macaques (Fischer et al. 1995) and chacma baboons (Fischer et al. 2001a). Given that these two species differ in how they perceive the graded variation in their calls (Fischer 1998; Fischer et al. 2001b), the graded structure of chirps presents an opportunity to further our more general understanding of how signal receivers respond appropriately to acoustically similar alarm calls produced in situations requiring incompatible escape behaviours.

## Playback experiments

### Experimental protocol

To test whether green monkey leopard chirps function referentially in that they (and not snake chirps) elicit leopard appropriate responses, and to investigate whether these responses are additionally influenced by supporting and conflicting contextual cues (presence of a leopard or snake simulated by a predator model), we used a within-subjects prime and probe playback design, with each of ten experimental subjects experiencing three un-primed and four primed conditions (Table 2). We balanced the order in which playback stimuli were presented and included call sequence as a fixed variable within statistical analyses.

**Table 2:** Description of the seven experimental conditions making up the prime and probe experimental design

Condition	Prime	Probe
1	None	Control
2	None	Leopard chirp
3	None	Snake chirp
4	Leopard model	Leopard chirp
5	Leopard model	Snake chirp
6	Snake model	Leopard chirp
7	Snake model	Snake chirp

Subjects were provisioned prior to playback experiments to position them on the ground at 8–15 m from a playback speaker that was hidden from sight behind a natural obstacle at a height of 1–1.5 m. Playback stimuli were broadcasted using a Marantz PMD-661 solid-state recorder connected to a loudspeaker (David Active, Visonik, Berlin), with maximum amplitude set within the range of natural calling behaviour (60–85 dB at 10 m from source, measured using a Voltcraft 322 sound level metre). For primed conditions, predator models were presented using the same protocol as described in Online Resource 1. When all alarm calling had stopped, a stop clock was started and a playback experiment was carried out as soon as possible within a 1-h time window. Subjects were played the chirp calls of an individual from the same group as themselves, and playbacks were carried out only when this individual was out of view. Experiments carried out on each subject were separated by ≥7 days, and a maximum of 3 playbacks (including a single leopard prime and/or a single snake prime) were carried out each week within a single group. Experiments were discarded if the wrong subject was filmed (2 cases), if the subject responded to a different stimulus prior to model presentation (1 case) or if there were technical problems with the equipment (4 cases).

### Behavioural analysis

Behavioural responses of subjects were filmed, videos were imported into Adobe Premiere CS4 and frame-by-frame analysis was used to score the subject’s behaviour as rest, bipedal, terrestrial displacement or arboreal displacement at 0.2 s intervals as described in the section of this manuscript looking at behavioural responses to predators. Video analysis started with the onset of the playback stimuli and continued for a period of 30 s. At the end of these 30 s, maximum height of the subject was recorded as 0 m, <2 m or >2 m. All subjects that responded with arboreal displacement did so immediately following initiation of the playback (subject in tree within 1.42 ± 0.75 s), and the time a subject spent arboreal was also measured, from when the subject entered a tree until the time when the subject returned to the ground. All videos were re-assessed by two condition-naive observers, and there was a high level of inter-observer reliability (intra-class correlation coefficient = 0.996).

### Statistical analysis

To ensure that subjects’ were responding to playbacks as a result of the call type and not the playback procedure itself, we ran a GLMM with binomial error structure and logit link function to model the likelihood that a subject would respond to a test versus control stimuli with any of bipedalism, terrestrial or arboreal displacement. Stimulus type (un-primed chirp or birdsong) was entered as the test predictor, playback order was entered as the control predictor (both as fixed effects) and subject identity was entered as a random effect. A likelihood ratio test was used to compare the full model with a null model, which retained all variables except stimulus type.

To assess the effect of call type and context on whether subjects would respond with a leopard-typical response, we ran a second GLMM with binomial error structure and logit link function to test differences in subjects’ propensity to climb >2 m into a tree. A third GLMM was run to assess whether call type or context would affect the amount of time individuals spent in a tree immediately after a playback experiment. We initially transformed the time that individuals spent in a tree into ordinal data, and used a Poisson error structure to model differences, but because the data were still overdispersed, we subsequently used a binomial error structure and logit link function to look at whether individuals stayed arboreal for longer than 200 s or not. We included call type (leopard or snake chirp), context (no prime, snake prime or leopard prime) and the interaction between the two as test predictors (fixed effects). Playback order was included as a control predictor (fixed effect), and subject identity and caller identity were included as random effects. We established the significance of the full model as compared to the null model (lacking all test predictors), and the full model as compared to reduced models (lacking the interaction and/or lacking the interaction and a test predictor) using a likelihood ratio test. Variance inflation factors were derived using the vif function of the R-package car (Fox and Weisberg 2011) and indicated that collinearity was not an issue. All models were fitted in R using the function lmer of the R-Package lme4.

### Results and discussion

**Fig. 4:**
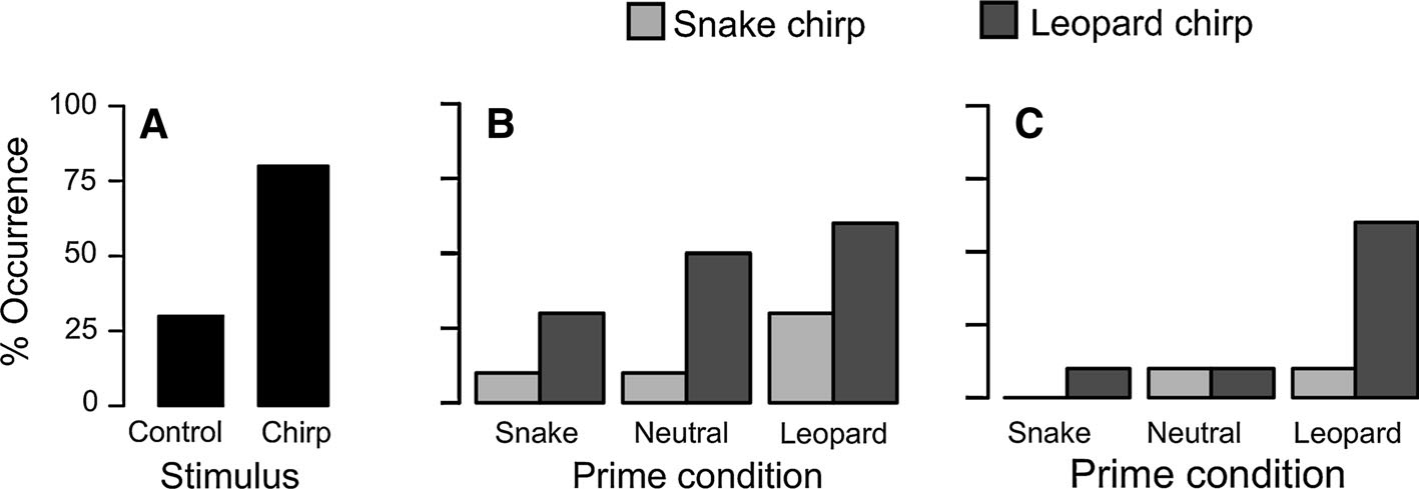
*Bar graphs* illustrating the percentage of trials in which subjects **a** responded to playbacks of control (*n* = 10) and chirp (*n* = 20) stimuli; **b** climbed to > 2 m within 30 s of hearing the playback stimuli, and **c** stayed > 200 s in a tree subsequent to hearing the playback stimuli. For playback experiments, *n* = 10 for all conditions

Subjects were significantly more likely to respond to playbacks of chirps than playbacks of bird calls (likelihood ratio test: *χ* ^*2*^ = 7.76, *df* = 1, *P* < 0.01, Fig. 4a). Behavioural responses to playbacks of chirp calls are thus due to signallers responding to the acoustic features of chirp calls, and not to some aspect of the playback process. In tests of whether subjects climbed to >2 m in a tree, the full model explained significantly more variation than the null model (*χ* ^*2*^ = 12.21, *df* = 5, *P* < 0.05), although only the effect of call type (with subjects climbing higher into a tree after hearing leopard than snake chirps; *χ* ^*2*^ = 8.17, *df* = 1, *P* < 0.01), but not prime stimulus (*χ* ^*2*^ = 3.28, *df* = 2, *P* = 0.19), was significant (Fig. 4b). In tests of the amount of time subjects spent in a tree immediately subsequent to playback experiments, the full model also explained significantly more of the variation than the null model (*χ* ^*2*^ = 14.44, *df* = 5, *P* < 0.05), but this time this effect was due not only to a significant effect of call type (*χ* ^*2*^ = 4.90, *df* = 1, *P* < 0.05), with subjects spending longer in a tree after hearing leopard than snake chirps, but also to a significant effect of context (*χ* ^*2*^ = 7.41, *df* = 2, *P* < 0.05) with subjects spending more time in a tree after being primed with a leopard model, Fig. 4c).

That subjects were more likely to climb >2 m into a tree in response to leopard chirps than to snake chirps irrespective of contextual cues suggests that green monkeys discriminated between graded variants of this alarm call and responded more often to leopard chirps as if a leopard were present. Given that the number of chirp units and the inter-unit duration between chirp units was kept constant between paired playbacks, this ability to discriminate between calls is apparently due to differences in call structure. At the same time, the structural similarity of the two chirp types suggests that differences in behavioural response are unlikely to be explained exclusively by unconditioned reactions to the acoustic properties of a call. Instead, it is likely that subjects’ responses are the result of a learnt association, which could be underpinned by subjects associating the call with the external referent (leopard) or with the emotional response experienced by listeners via “affect conditioning” (Owren and Rendall 1998). At the ultimate level, it has been claimed that selection pressures act on receiver’s “data-acquisition mechanisms” (motivation, attention and rule learning, Lotem and Halpern 2012) to enable them to process the relevant acoustic cues and to respond appropriately. In accordance with this, selection may well have acted on the perceptual system of the green monkey to enable them to both recognise the small but biologically relevant differences existing between chirps, and perhaps also to form the relevant associations faster whilst experiencing a high level of arousal. An alternative explanation is that signal receivers may respond more strongly to leopard chirps because they are a more urgent call associated with contexts of higher caller arousal, a point supported by the finding that leopard chirps differ from snake chirps in parameters that frequently indicate higher caller arousal (Briefer 2012).

It is important to note that despite their apparent ability to differentiate to some extent between leopard and snake chirps, green monkeys, like vervets (Seyfarth et al. 1980b), do sometimes respond with inappropriate escape behaviour. This could be explained by the unequal costs of inappropriate responses (Godfrey-Smith 1991). For example, the high cost to individuals of not climbing a tree when a leopard is present versus the smaller cost of climbing a tree when a snake is present could have led to a bias of green monkeys attributing chirps to leopard presence when the signal is ambiguous in terms of its association with either a leopard or snake. That green monkeys in this study sometimes responded to snake chirps by climbing into a tree support this hypothesis, but the finding that subjects did not always respond to leopard chirps by climbing into a tree does not. It could also be that climbing into a tree is, in some cases, an adaptive response to a snake, and/or that other contextual cues are required for listeners to attribute meaning to their chirps with a high degree of certainty.

In this study, priming with a leopard model increased the chances of a subject responding to both leopard and snake chirps with a leopard-typical response (climbing >2 m into a tree), but this effect was small and did not reach significance. It is possible that the effects of context on such responses are subtle and were not picked up with the small sample size of this study. It could also be that the contextual prime (presented up to an hour before the playback of calls) became less relevant over longer time intervals. This could explain differences between this and another study in which context was found to affect Diana monkey responses to Guinea fowl alarm calls, as contextual primes in that study were given just 5 min prior to the broadcasting of alarm calls (Zuberbühler 2000). However, the behaviour of vervet monkeys indicates that they remember the location of a predator for at least 2 h after seeing it (Cheney and Seyfarth 1992b). It is known that vervets frequently respond to playbacks of predator-specific alarm calls by looking towards the speaker and scanning the surrounding environment before responding with escape behaviour (Seyfarth et al. 1980b), and laboratory studies suggest that a subject’s surroundings can affect how conditioned stimuli are perceived (Pearce and Bouton 2001). It is therefore possible that contextual cues present at the time of call perception (e.g. the behaviour of group members) were more salient than the recent sighting of a predator and had a larger influence on listeners’ initial attribution of meaning and immediate response.

In contrast to the lack of an effect of priming context on immediate responses, we did find that both call type and context had an effect on the length of time that subjects remained in a tree following a playback. Specifically, subjects stayed longer in a tree after hearing leopard chirps for the most part only after having been primed with a leopard model. The lack of an interaction between call type and context is likely due to the fact that GLMMs lack the power to identify interactions when sample sizes are small (R. Mundry, personal communication). It is therefore possible that an individual’s prior knowledge was incorporated to refine meaning attribution at a later point in time, leading to the individual staying longer in a tree when both vocal and contextual cues pointed to a leopard being present. Alternatively, signal perception may involve separate meaning attribution and decision making processes, each of which may vary based on additional contextual cues (Fischer 2013). If this is the case, it is possible that staying longer in a tree was the result not of a difference in meaning attribution, but of a difference in a subsequent decision making process.

## Conclusion

Adult green monkeys respond to graded differences in the vocal structure of their chirp calls, on average, with an appropriate anti-predator escape behaviour. The fact that acoustic cues were insufficient to elicit appropriate responses in all individuals, however, suggests that context likely does play a role in how green monkeys attribute meaning to these calls, but that a receiver’s prior knowledge may play a role in delayed rather than immediate attribution of meaning. Studies that systematically test, under natural settings, whether different types of contextual cues are integrated as a part of meaning attribution and/or feed into a separate decision making process will be particularly useful in furthering understanding of the flexibility of cognitive mechanisms underlying call perception in animals.

## Electronic supplementary material

Below is the link to the electronic supplementary material.

Supplementary material 1 (DOCX 10261 kb)
