# Supplementary material for: Meaning attribution in the West African green monkey: influence of call type and context
Source: Anim Cogn. 2013 Jul 12;17(2):277–86. doi: 10.1007/s10071-013-0660-9 (PMC3920029; doi:10.1007/s10071-013-0660-9)
Supplement: Supplementary file 1 — Supplementary material 1 (DOCX 10261 kb) [file 10071_2013_660_MOESM1_ESM.docx]

**Animal Cognition**

Meaning attribution in the West African green monkey: influence of call type and context

Tabitha Price^1,2*^, Julia Fischer^1,2^

^1^ Cognitive Ethology Lab, German Primate Center, Germany

^2^ Courant Research Centre for the Evolution of Social Behaviour, Georg August University of Goettingen,

Germany

*Contact: [tprice@dpz.eu](mailto:tprice@dpz.eu)

**Predator simulations and modes of presentation**

Snake models consisted of one rubber snake painted to resemble a black mamba and two python models. Leopard models consisted of three life-size stuffed toy leopards and one leopard print fur cover. For one experiment looking at behavioural responses to predators, an audio presentation of leopard growls was used. Pictures of a selection of these predator models are shown below.

To simulate snake presence, a snake model was hidden in the undergrowth 11-26m from the subject and drawn across the ground using translucent fishing twine and a custom built pulley system. To simulate leopard presence, a leopard model, or a person draped with leopard cover was hidden 7- >60m from the subject until the time of the experiment when the model or person was revealed. For one experiment an audio speaker was concealed 20m from the subject and leopard growls (recorded by TP from a local captive leopard) were broadcast.

**Snake models**

**Leopard model**


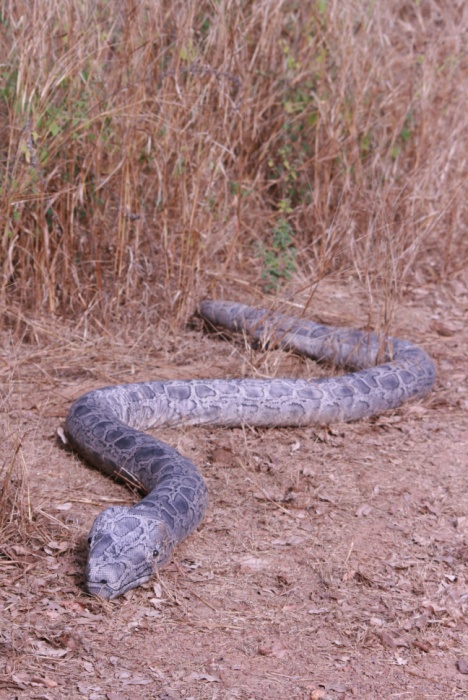


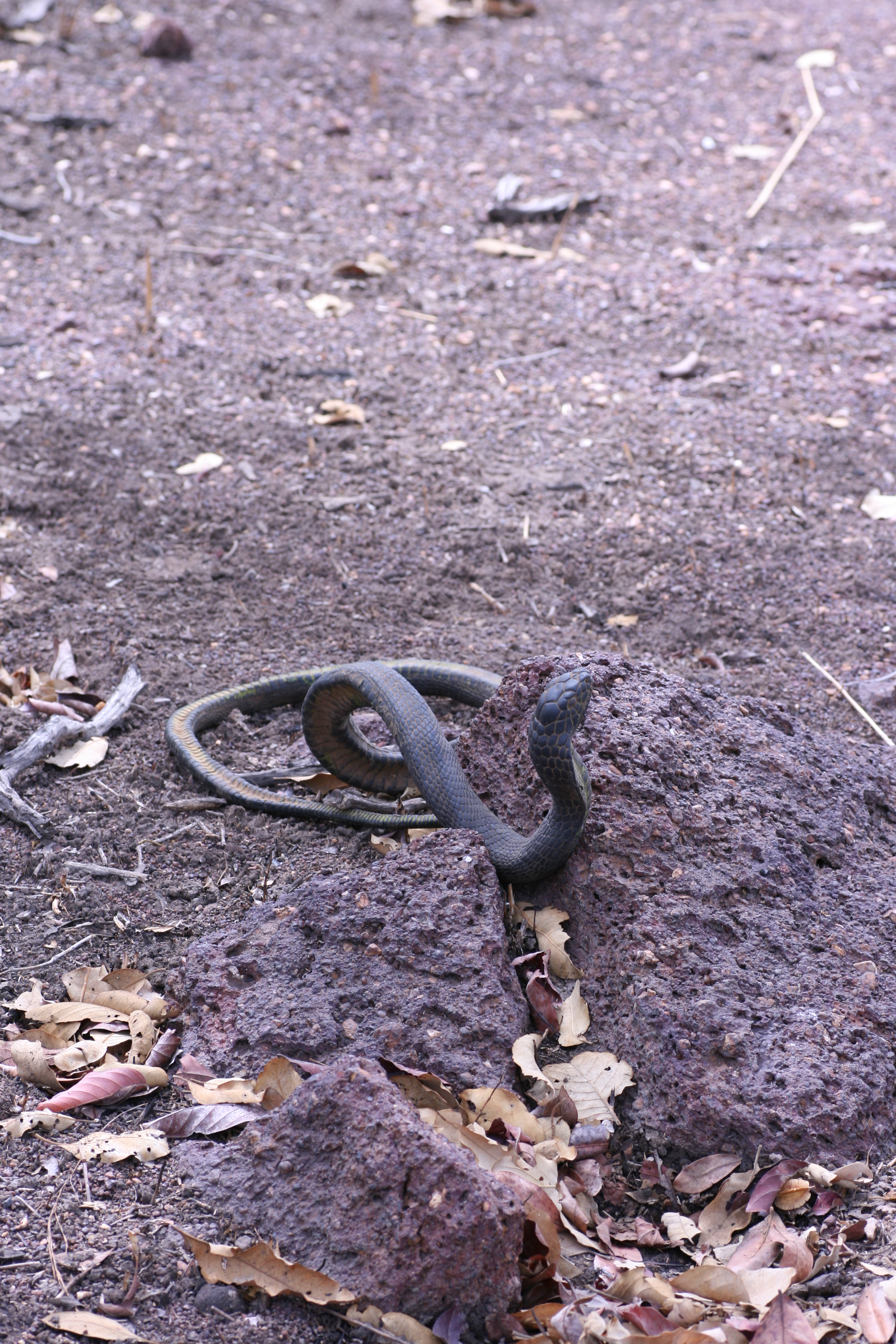

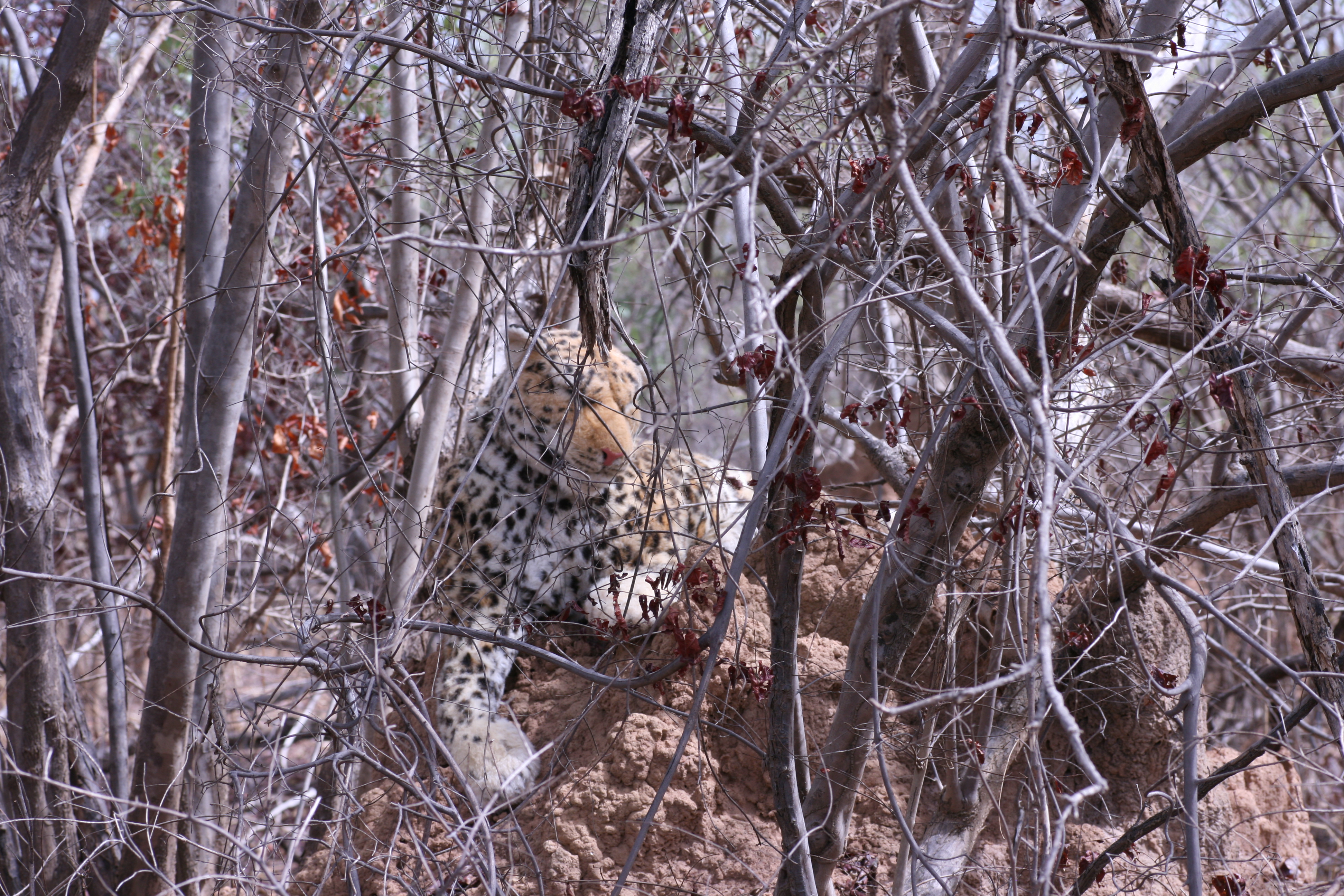


**Chirp playback stimuli: Statistical analysis**

Table of transformations applied to the raw data of acoustic parameters prior to LDA

| Parameter | Transformation |
| --- | --- |
| Duration | ^-0.2 |
| Frequency range | ^0.1 |
| Jump | Log |
| Peak frequency deviation | Log |
| Second quartile_1 | ^2 |
| Second quartile_2 | Square root |
| Second quartile_3 | Log |
| Second quartile_4 | Log |
| Third quartile_1 | ^-0.6 |
| Third quartile_3 | ^-0.6 |
| Third quartile_4 | ^2 |
| Peak frequency_2 | Log |
| Peak frequency_3 | Log |
| Peak frequency_4 | Log |
